# External validation of models for predicting risk of colorectal cancer using the China Kadoorie Biobank

**DOI:** 10.1186/s12916-022-02488-w

**Published:** 2022-09-08

**Authors:** Roxanna E. Abhari, Blake Thomson, Ling Yang, Iona Millwood, Yu Guo, Xiaoming Yang, Jun Lv, Daniel Avery, Pei Pei, Peng Wen, Canqing Yu, Yiping Chen, Junshi Chen, Liming Li, Zhengming Chen, Christiana Kartsonaki

**Affiliations:** 1 Clinical Trial Service Unit & Epidemiological Studies Unit (CTSU), Nuffield Department of Population Health, Big Data Institute Building, Roosevelt Drive, University of Oxford, Oxford, UK; 2 Department of Surveillance and Health Equity Science, American Cancer Society, Atlanta, GA USA; 3 Medical Research Council Population Health Research Unit (MRC PHRU), Nuffield Department of Population Health, Big Data Institute Building, University of Oxford, Old Road Campus, Oxford, OX3 7LF UK; 4 Fuwai Hospital, Chinese Academy of Medical Sciences, Beijing, 102308 China; 5 Department of Epidemiology and Biostatistics, School of Public Health, Peking University, 38 Xueyuan Road, Beijing, 100191 China; 6 Chinese Academy of Medical Sciences, Building C, NCCD, Shilongxi Rd., Mentougou District, Beijing, 102308 China; 7 Maiji CDC, No. 29 Shangbu Road, Maiji, Tianshui, 741020 Gansu China; 8 National Center for Food Safety Risk Assessment, 37 Guangqu Road, Beijing, 100021 China

**Keywords:** Cancer epidemiology, Colorectal cancer, Risk prediction models, External validation

## Abstract

**Background:**

In China, colorectal cancer (CRC) incidence and mortality have been steadily increasing over the last decades. Risk models to predict incident CRC have been developed in various populations, but they have not been systematically externally validated in a Chinese population.

This study aimed to assess the performance of risk scores in predicting CRC using the China Kadoorie Biobank (CKB), one of the largest and geographically diverse prospective cohort studies in China.

**Methods:**

Nine models were externally validated in 512,415 participants in CKB and included 2976 cases of CRC. Model discrimination was assessed, overall and by sex, age, site, and geographic location, using the area under the receiver operating characteristic curve (AUC). Model discrimination of these nine models was compared to a model using age alone. Calibration was assessed for five models, and they were re-calibrated in CKB.

**Results:**

The three models with the highest discrimination (Ma (Cox model) AUC 0.70 [95% CI 0.69–0.71]; Aleksandrova 0.70 [0.69–0.71]; Hong 0.69 [0.67–0.71]) included the variables age, smoking, and alcohol. These models performed significantly better than using a model based on age alone (AUC of 0.65 [95% CI 0.64–0.66]). Model discrimination was generally higher in younger participants, males, urban environments, and for colon cancer. The two models (Guo and Chen) developed in Chinese populations did not perform better than the others. Among the 10% of participants with the highest risk, the three best performing models identified 24–26% of participants that went on to develop CRC.

**Conclusions:**

Several risk models based on easily obtainable demographic and modifiable lifestyle factor have good discrimination in a Chinese population. The three best performing models have a higher discrimination than using a model based on age alone.

**Supplementary Information:**

The online version contains supplementary material available at 10.1186/s12916-022-02488-w.

## Background

Colorectal cancer (CRC) is the third most diagnosed cancer and the second most common cause of cancer-related death worldwide [1]. In China, CRC incidence and mortality have been steadily increasing over the last decades [2]. As a result, strategies to identify those at higher risk are needed in China to improve early detection of CRC and reduce the burden of disease. In many high-income countries, decisions around screening for CRC are based on age alone. The US Preventative Services Task Force recommends screening for CRC in all adults aged 50–75 years [3]. In the UK, all adults aged 56–74 are offered a faecal immunochemical test (FIT) every 2 years and those with abnormal FIT results are referred to colonoscopy, the gold-standard test for diagnosing CRC [4]. These screening programmes have shown to be effective in reducing mortality of CRC [5, 6]. In China, there is currently no nationwide screening programme [7, 8]. While there is some screening regionally and for those at high risk (defined as having a first degree relative with colorectal cancer, history of cancer, or history of bowel conditions), it is not standardised and uptake is limited [9]. It is not clear whether using age as the only factor to screen for CRC would be an effective screening strategy in China. Moreover, it would be useful to know whether an age-based screening strategy could be improved upon by adding other modifiable risk factors to enhance prediction of CRC. Prognostic risk prediction models, based on easily obtainable demographic, medical history, and lifestyle variables, can be used to stratify the population to identify high-risk individuals, guide referral to screening, and motivate behavioural changes that could reduce risk [10].

Previous systematic reviews have identified risk prediction models for CRC developed in various populations, but they have not been systematically externally validated in a Chinese population [11]. In this study, we aimed to assess the performance of risk scores in predicting CRC using the China Kadoorie Biobank (CKB), one of the largest and geographically diverse prospective cohort studies in China. Specifically, we aim to externally validate published risk scores for predicting CRC based on lifestyle and demographic information and determine how these models compare to using an age threshold alone as a screening strategy.

## Methods

### Selection of risk prediction models

**Fig. 1:**
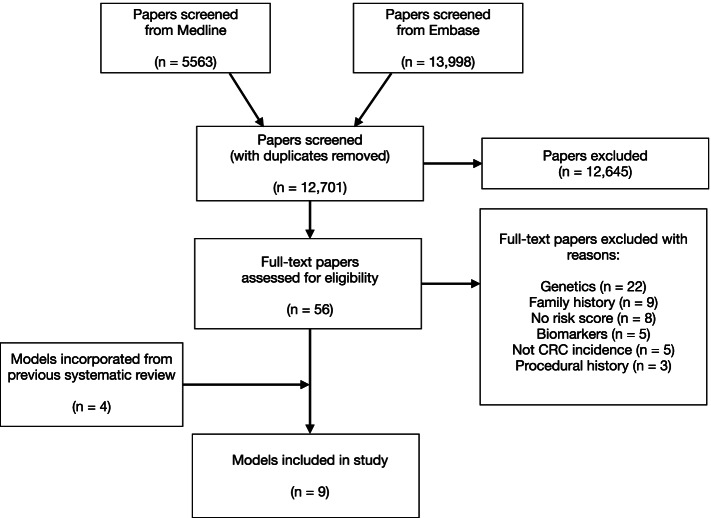
PRISMA flow diagram of the updated systematic review of risk prediction models for CRC

We identified nine risk prediction models for either CRC, colon cancer, or rectal cancer that met our inclusion criteria (Fig. 1) by updating a previous systematic review from November 2016 to June 2021 (Additional File 1: Page S1) [11]. We excluded 12,645 articles based on their title and abstract, screened 56 full-text articles. From the full text, we excluded 22 models that included genetic or biochemical biomarkers, nine that included family history, and eight articles that did not include a risk score but described risk factor associations with CRC (Fig. 1). Five models assessed prognosis of those diagnosed with CRC and were excluded, and three models were excluded for containing procedural variables. Eight articles were included in our study, including a total of nine models (Ma article developed two different models [12]). We performed an external validation of these risk models following the TRIPOD (Transparent Reporting of a multivariable prediction model for Individual Prognosis Or Diagnosis) guideline (Additional File 1: Page S2) [13].

### Study population

Data from the China Kadoorie Biobank (CKB), a large, prospective population-based cohort in China, were used to externally validate the risk models. The details of the CKB design, survey methods, and population characteristics are described elsewhere [14]. In brief, 512,726 participants aged 30–79 years were recruited into the study between 2004 and 2008 from 10 geographically defined localities (5 urban and 5 rural) in China. Central ethical approvals were obtained from Oxford University and the China National Centre for Disease Control and Prevention (CDC). Approvals were also obtained from institutional research boards at the local CDCs in the 10 areas. At local assessment centres, participants completed an interviewer-administered laptop-based questionnaire on sociodemographic characteristics, smoking, alcohol consumption, diet, physical activity, personal and family medical history, and current medication. A range of physical measurements were recorded by trained technicians, including height, weight, hip and waist circumference, blood pressure, and heart rate, using calibrated instruments with standard protocols. A description of how the variables in CKB were ascertained is given in Additional File 1: Page S3 [15, 16].

### Follow-up for cancer incidence and mortality

The vital status of each participant was determined periodically through China CDC’s Disease Surveillance Points (DSP) system and national health insurance system, supplemented by regular checks against local residential and administrative records and by annual active confirmation through street committees or village administrators. In addition, information about major diseases and any episodes of hospitalisation was collected through linkage, via each participant’s unique national identification number, with cancer registries, national health insurance claims databases, and death registries. Data on colorectal cancer incidence and mortality was available for each participant up to December 31, 2017. All death, diagnosis, or hospitalisation events were coded using International Classification of Disease 10^th^ Revision (ICD-10) by trained staff who were blinded to baseline information [14]. Information on cancer histological subtypes was also collected for a subset of the cases through cancer registries or reviews of hospital medical notes as part of the ongoing outcome adjudication for major diseases.

### Risk model prediction variables and outcome variables

For each risk factor, we used data collected from the baseline questionnaire. Variables from the CKB dataset were matched as closely as possible to the variables used in each model. If there was not an exact equivalent, proxy variables were derived. Full details of the risk factor definitions, how they were operationalised in the CKB dataset, and how missing data were handled are given in Additional File 1: Page S4 [17–19]. The outcome for each risk model was a composite outcome based on incidence or death from colorectal cancer (ICD C18-20), colon cancer (ICD C18), and rectal cancer (C19-20), as well as right-sided colon cancer (ICD 18.0–18.3) and left-sided colon cancer (ICD C18.5–18.7) using data from linked cancer registries, health insurance records, and death registries.

### Statistical methods

The discrimination and calibration of risk prediction models were computed. Participants were followed-up from study entry to diagnosis or death from CRC, loss to follow-up, death from other causes, or 10 years since study entry, whichever occurred first. Because the aetiology of site-specific CRCs is hypothesised to differ, model discrimination was also assessed in cancers of the colon and rectum separately, as well as in right- and left-sided colon cancer [20, 21]. Discrimination was also assessed separately in males and females (even if the model was developed only from males), urban and rural populations (because the increasing incidence of CRC in China has been hypothesised to be linked to more “western” urban lifestyles [22, 23]), and in those age 56 years or older versus younger than age 56 to determine how the full models perform in older and younger participants. Discrimination among the nine models was also compared to a model based on age alone, by comparing to the national UK colorectal screening age cut-offs (adults between 56 and 74 are screened using FIT every 2 years). A model was fit with the only explanatory variable being age 56 or older versus younger than age 56 to determine how the multivariable models perform compared to a model just using an age cut-off. The primary outcome was incidence or mortality from CRC, and the discriminative capability of the models was compared using receiver operating characteristic curves (ROC) and areas under the ROC (AUC). Sensitivity, specificity, and both positive and negative predictive values were computed for the 10% and 25% of participants with the highest risk of CRC, as determined by each model. In addition, the difference in ROC analyses between the age only model and the models that included lifestyle risk factors was quantified using the Delong method [24].

Calibration was assessed graphically for models that included estimations of absolute risk of CRC at different risk score levels in the published article presenting the model. Observed risk was plotted against expected risk of developing CRC over the 10-year period in groups based on quantiles of expected risk and the slope and intercept were estimated. Most models predicted risk over 10 years, other than the Driver [25] model, which predicted risk over 20 years and required converting the predicted risk to over 10 years by assuming an exponential distribution. To re-calibrate the models, the predicted scores were split at their deciles and the slope and intercept of the observed risk plotted against expected risk graph was computed. Each score value (corresponding to a decile of risk) was multiplied by the slope and the intercept was added, to produce a new re-calibrated set of estimates of absolute risk of CRC.

Several sensitivity analyses were carried out. Discrimination analyses were performed by fitting a Cox regression and comparing the C-statistic, which considers the time of CRC onset, to the ROC analysis, which is based on a binary outcome variable. The rationale for using the ROC analysis is both to compare results to existing literature, and to compare model performance in different cohorts. Finally, because the aspirin variable is only available for a subsection of the population in CKB (those with a history of coronary heart disease), we removed the aspirin variable from the two models that contained it (Imperiale [26] and Hong [27] models) and compared their performance.

Analyses were done using R version 3.6.3 and packages pROC (version 1.18), stringi (version 1.7.5), tidyverse (version 1.3.1), and table 1 (version 1.1).

**Table 1:**
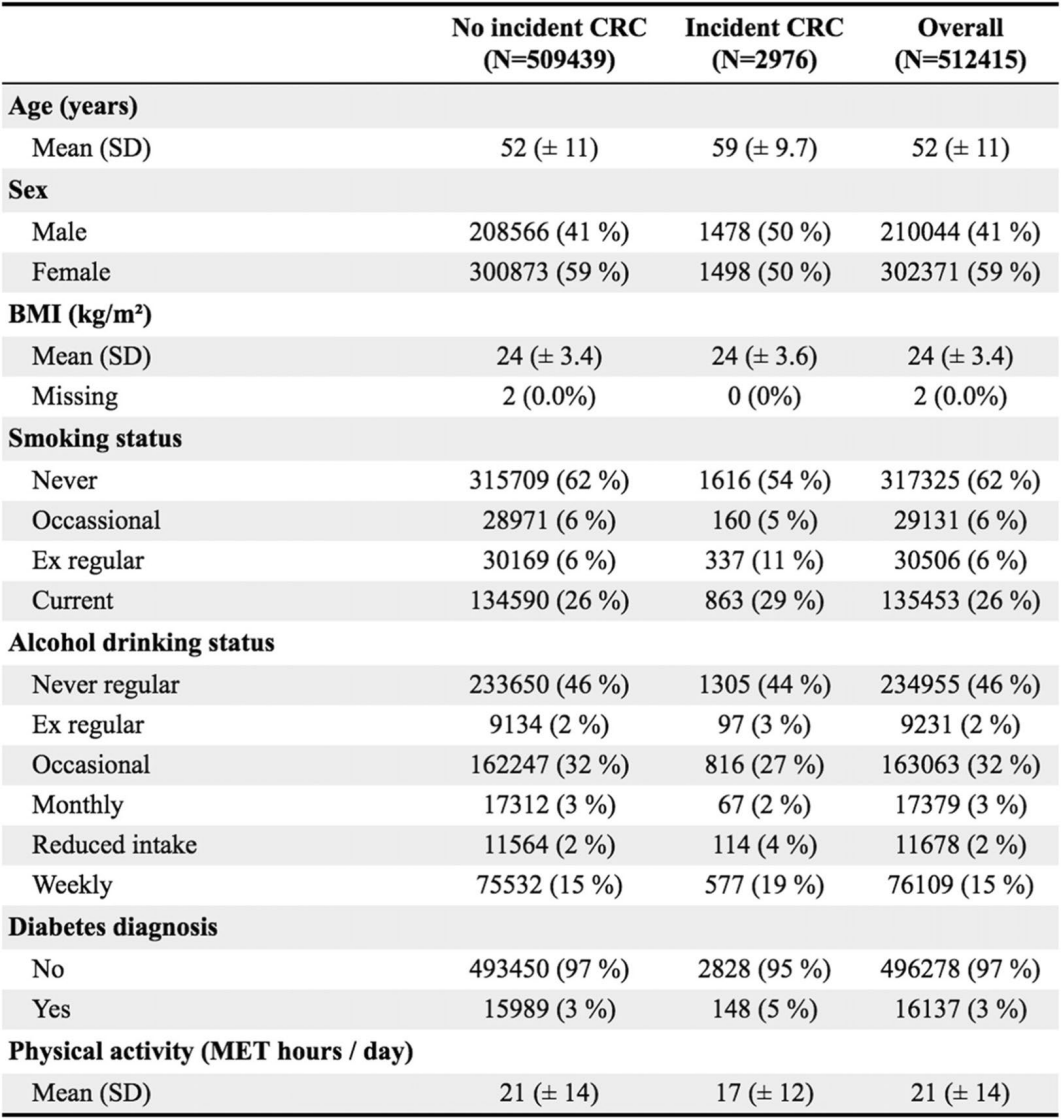
Characteristics of participants in the China Kadoorie Biobank cohort up to 10 years of follow-up used for external validation. Distribution of variables are shown between those with without incident colorectal cancer

## Results

Because most risk models predict the 10-year risk of developing CRC, we included up to 10 years of follow-up for each participant. The 311 participants previously diagnosed with intestinal cancer, and two participants with missing BMI, were excluded. This resulted in 512,415 participants included in the primary analyses. Among those, there were 2976 cases of incident CRC (which includes cancer anywhere from the caecum to the rectum, including cancer in both the colon and rectum), 1720 cases of incident colon cancer, and 1772 cases of incident rectal cancer. Characteristics of participants with and without incident CRC are given in Table 1. Those with CRC were more likely to be older, male, current or ex-regular smokers, weekly alcohol drinkers, have a diagnosis of diabetes, and do less physical activity.

### Characteristics of included models

Of the nine included models, all had CRC as the main outcome and two articles, including three models (Driver [25], Ma Point [12], Ma Cox [12]), additionally considered colon or rectal cancer as separate outcomes. Information about these models, including the study size, variables used in the model, and cancer outcomes included, are given in Table 2. All articles published either a point score, Cox proportional hazards model, or a logistic regression, other than the Ma article, which had both a point score and Cox model. Four models (Driver [25], Ma Point [12], Ma Cox [12], and Guo [28]) were developed only in a male population; the rest were developed in both male and female populations. Two models (Guo [28] and Chen [29]) were developed in Chinese populations. Five models were developed using < 500 cases of CRC, the two Ma [12] models used 543 cases, the Hong [27] model was developed from 1117 cases, and Aleksandrova [30] from 3645 cases of CRC.

**Table 2:** Details of the development of the risk scores used for the external validation

Author (year)	Study size (cases of CRC)	Model type	Country	Cancer outcome	Model evaluated in M or F
Driver (2007) [25]	21,581 (485)	Logistic regression	USA	CRC, C, R	M
Ma Point (2010) [12]	18,256 (543)	Point score	Japan	CRC, C, R	M
Ma Cox (2010) [12]	18,256 (543)	Logistic regression	Japan	CRC, C, R	M
Guo (2020) [28]	92,923 (353)	Point score	China	CRC	M
Chen (2014) [29]	905 (38 CRC; 100 AP)	Point score	China	CRC	Both
Betes (2003) [31]	2210 (270)	Point score	Spain	CRC	Both
Aleksandrova (2021) [30]	255,482 (3645)	Logistic regression	Europe	CRC	Both
Imperiale (2021) [26]	3025 (284)	Logistic regression	USA	CRC	Both
Hong (2017) [27]	21,762 (1117)	Logistic regression	Korea	CRC	Both

Variables included in the risk scores are given in Table 3. Age was included in all models and all models (other than Guo [28] and Aleksandrova [30]) included either sex or BMI. The other most frequently included variables were smoking, alcohol, and physical activity. Three models included dietary variables and two included aspirin intake. Details for the full equations of the risk models are given in Additional File 1: Page S5.

**Table 3:** Factors included in the risk scores used for the external validation

	**Variables included in score**							
**Author (year)**	**Age**	**Sex**	**BMI**	**Smoking**	**Alcohol intake**	**Diabetes**	**Physical activity**	**Other**
Driver (2007) [25]	Yes	No	Yes	Yes	Yes	No	No	
Ma Point (2010) [12]	Yes	No	Yes	Yes	Yes	No	Yes	
Ma Cox (2010) [12]	Yes	No	Yes	Yes	Yes	No	Yes	
Guo (2020) [28]	Yes	No	No	No	Yes	Yes	No	Waist circumference, occupational sitting time
Chen (2014) [29]	Yes	Yes	No	No	No	No	No	History of coronary heart disease, egg intake, defecation frequency
Betes (2003) [31]	Yes	Yes	Yes	No	No	No	No	
Aleksandrova (2021) [30]	Yes	No	No	Yes	Yes	No	Yes	Waist circumference, body height, vegetable intake, dairy intake, processed meat intake, sugar, and confectionary intake
Imperiale (2021) [26]	Yes	Yes	No	Yes	Yes	No	Yes	Marriage, education, non-steroidal anti-inflammatory drugs (NSAID) use, metabolic syndrome, red meat, aspirin use
Hong (2017) [27]	Yes	Yes	No	Yes	Yes	No	No	Aspirin use

### Model discrimination

**Fig. 2:**
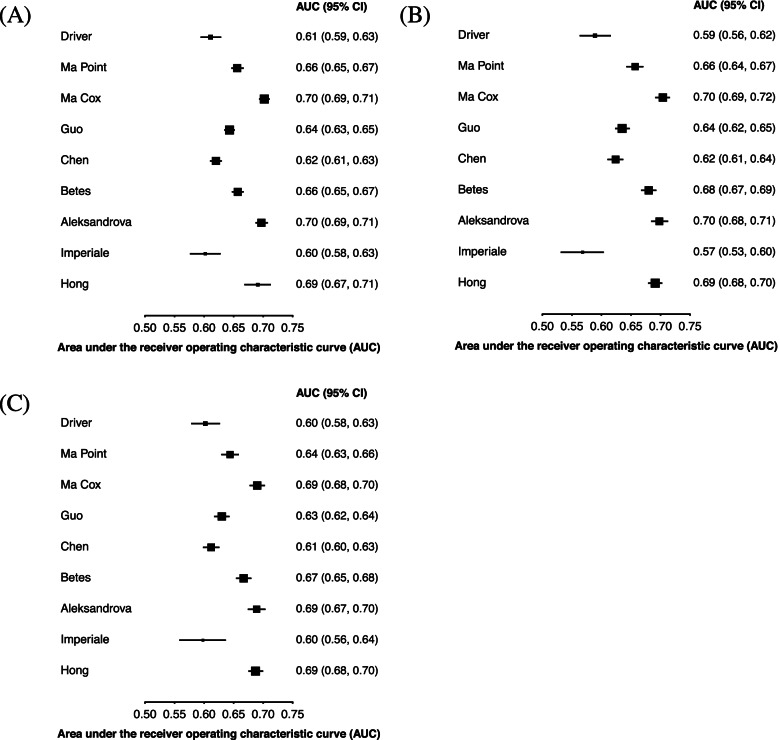
Model discrimination for 10-year risk of developing colorectal cancer. Area under the receiver operating characteristic curve for the risk models in **A** males and females, **B** males, and **C** females

Figure 2A shows the AUC for colorectal cancer in males and females and separated by sex (Fig. 2B for males and 2c for females). The three models with the highest discrimination (Ma Cox AUC 0.70 [95% CI 0.69–0.71]; Aleksandrova 0.70 [0.69–0.71]; Hong 0.69 [0.67–0.71]) include age, smoking, and alcohol in their models. In addition, Ma Cox [12] and Aleksandrova [30] included BMI or waist circumference and Hong [27] included sex. In contrast, Driver [25], Imperiale [26], and Chen [29] had the lowest discrimination (Driver AUC 0.61 [95% CI 0.59–0.63]; Imperiale 0.60 [0.58–0.63]; Chen 0.62 [0.61–0.63]). The age threshold model had an AUC of 0.65 [0.64–0.66], which was statistically significantly lower than the three best performing models when compared using the Delong method (*p* < 0.001 for the Ma Cox, Aleksandrova, and Hong model, respectively). In terms of sex differences, the Driver [25] and Imperiale [26] models performed better in females, the Hong [27] model performed the same, and all other models performed similarly by sex. Of the four models that were developed in males, all performed better in males except Driver [25]; however, four of the five developed in both males and females also performed better in males.

**Fig. 3:**
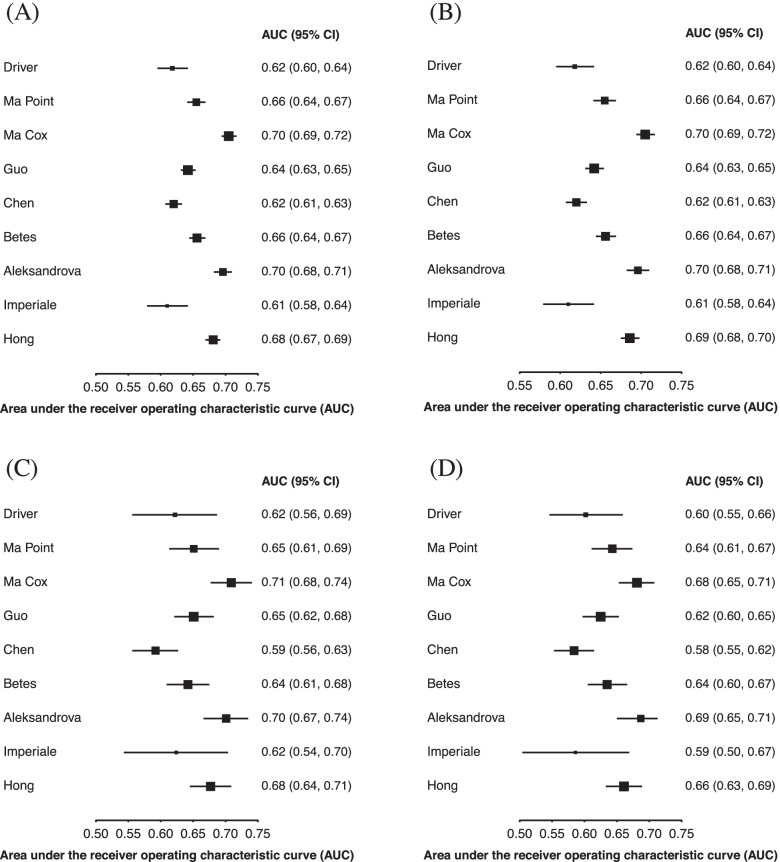
Model discrimination for 10-year risk of developing site-specific colorectal cancer. Area under the receiver operating characteristic curve for predicting **A** colon cancer, **B** rectal cancer, **C** right-sided colon cancer, and **D** left-sided colon cancer

**Fig. 4:**
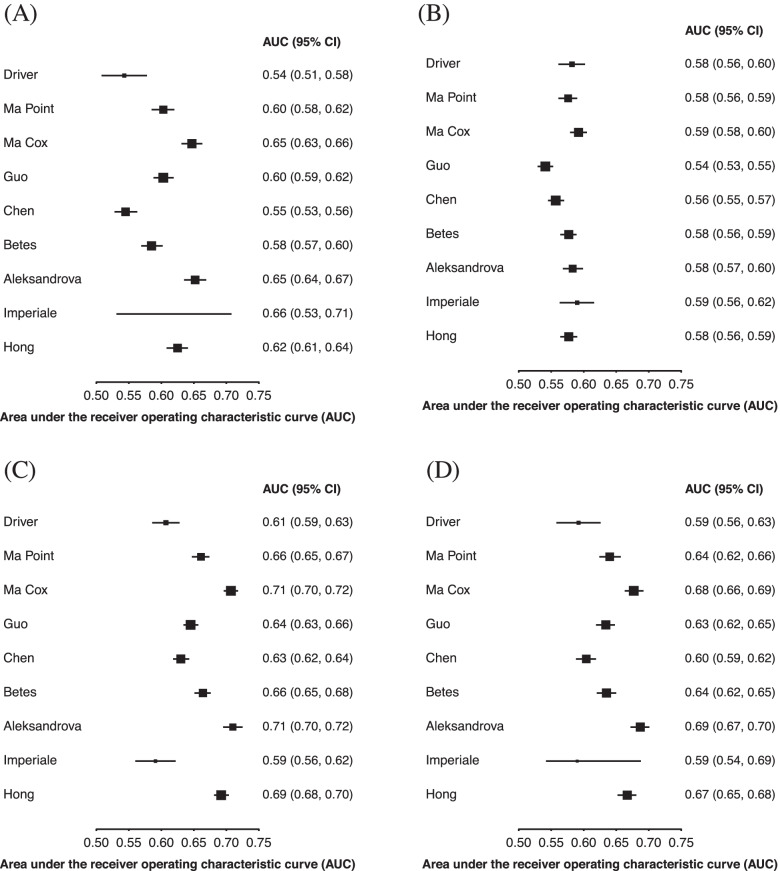
Model discrimination for 10-year risk of developing colorectal cancer by age and geographic location. Area under the receiver operating characteristic curve for the risk models in **A** younger participants (age < 56 years), **B** older participants (age ≥ 56), **C** participants from urban settings, and **D** participants from rural settings

Figure 3A shows colon cancer and Fig. 3B shows rectal cancer outcomes. The overall discrimination for colon cancer was similar compared to CRC (AUC range 0.61–0.70) and discrimination was generally similar for predicting rectal cancer (AUC range 0.59–0.69). The discrimination of models was generally similar for right-sided colon cancer, compared to the combined colon cancer outcome (Fig. 3C, A, respectively), but both were lower than the left-sided colon cancer outcome (Fig. 3D). Figure 4 shows the discrimination of the models in predicting CRC by comparing those younger than 56 to those aged 56 or older (Fig. 4A, B, respectively) as well as those in urban and rural environments (Fig. 4C, D, respectively). In general, models performed better in younger participants than in older ones; in older participants, all models had an AUC lower than 0.60, whereas in younger participants, six models had an AUC higher than 0.60. When comparing models evaluated on participants from urban and rural environments, all models performed better in urban environments than in rural ones, except for the Imperiale [26] model which performed the same in both environments.

Table 4 shows sensitivity, specificity, and positive and negative predictive values for the 10% and 25% of participants with the highest risk, predicted by each model. Among the 10% of participants with the highest risk, the Ma Cox [12] and Aleksandrova [30] models identified 25.9% and 25.2% of participants, respectively, that went on to develop CRC. In contrast, the Chen [29], Guo [28], and Driver [25] models only identified 6.8%, 7.6%, and 9.2% of those that went on to develop CRC. Among the 25% of participants with the highest risk, the Ma Cox [12] and Aleksandrova [30] models identified 52.7% and 51.8% of participants, respectively, that went on to develop CRC. In contrast, the Imperiale [26], Betes [31], and Driver [25] models only identified 32.7%, 35.5%, and 36.8% of those that went on to develop CRC. The specificity was above 90% for all models for the 10% of participants with the highest risk. The Hong [27] and Aleksandrova [30] models had the lowest specificity (90.1%) and the Guo [28] and Chen [29] models had the highest of (96.3% and 95.6%, respectively). Among the 25% of participants with the highest risk, the model specificity ranged from 75.1% in the Aleksandrova [30] and Hong [27] models to 84% in the Betes [31] model. The positive predictive values for the 10% with the highest risk ranged from 1.2% (Guo model) to 2.3% (Driver model). The negative predictive values for the 10% with the highest risk ranged from 99.0% (Driver and Imperiale models) to 99.6% (Aleksandrova model).

**Table 4:** Sensitivity, specificity, and positive and negative predictive value for 10% and 25% of participants with the highest risk

	Driver	Ma Point	Ma Cox	Guo	Chen	Betes	Aleksandrova	Imperiale	Hong
**Top 10%**									
Sensitivity	9.17	18.2	25.9	7.63	6.81	15.5	25.2	12.5	24.0
Specificity	96.1	92.1	90.1	96.3	96.5	94.7	90.1	92.7	90.1
PPV (%)	2.30	1.37	1.50	1.19	1.30	1.68	1.25	1.87	1.39
NPV (%)	99.0	99.5	99.5	99.4	99.4	99.5	99.6	99.0	99.5
**Top 25%**									
Sensitivity (%)	36.8	38.7	52.7	40.1	38.7	35.5	51.8	32.7	48.7
Specificity (%)	76.9	80.7	75.2	76.9	77.2	84.0	75.1	78.6	75.1
PPV (%)	1.60	1.20	1.22	1.00	1.12	1.28	1.03	1.67	1.13
NPV (%)	99.2	99.5	99.6	99.5	99.5	99.6	99.7	99.0	99.6

### Model calibration

**Fig. 5:**
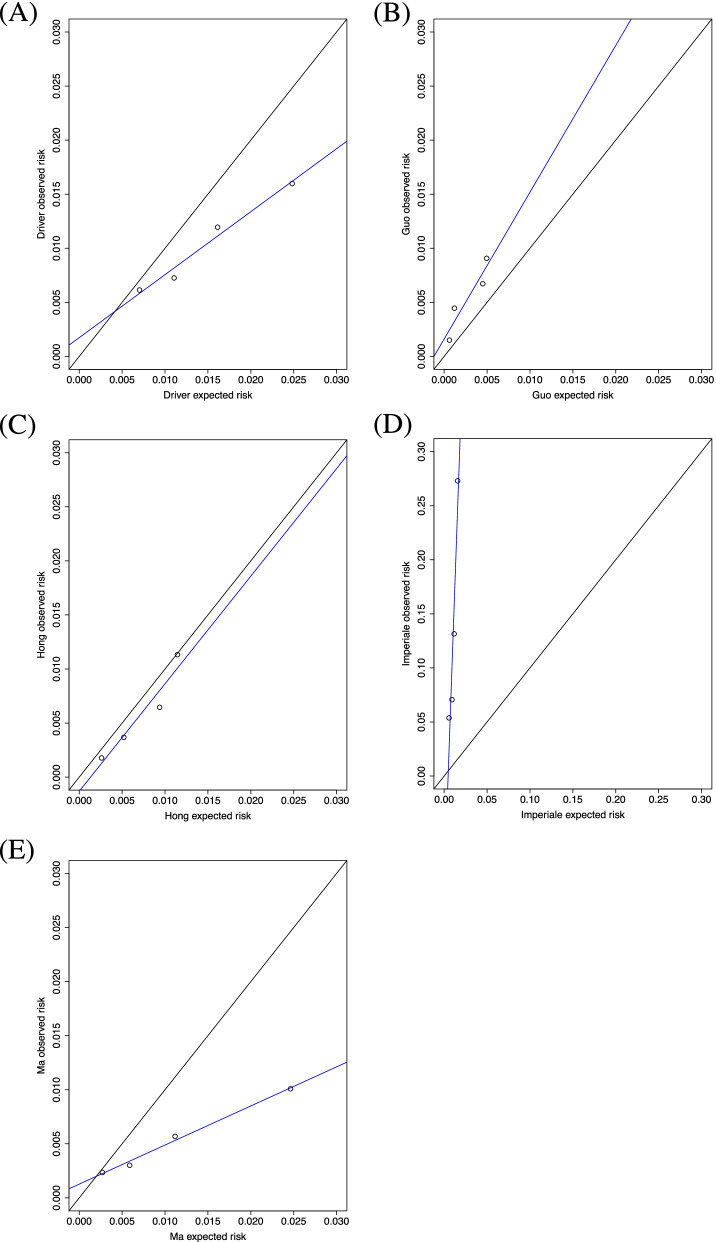
Model calibration curves of observed and expected 10-year risk of colorectal cancer in men and women. **A** Driver risk score, **B** Guo risk score, **C** Hong risk score, **D** Imperiale risk score, and **E** Ma Point risk score

Five models contained estimations of absolute risk of CRC in the published articles and their calibration could be assessed in CKB (Ma Point [12], Imperiale [26], Driver [25], Guo [28], and Hong [27]). Figure 5 shows the observed and expected 10-year risk of CRC for those models in males and females combined. Three models (Ma Point [12], Driver [25], and Hong [27] models) overestimated risk and two models (Imperiale [26] and Guo [28] models) underestimated risk, especially at higher levels of observed risk. Models were recalibrated and the slope and intercept were adjusted to match the observed and expected risks. The recalibrated expected 10-year risks for each model fitted to CKB data are given in Additional File 2: Table S1 and the recalibrated curves can be found in Additional File 3: Figure S1.

### Sensitivity analyses

The results from the sensitivity analyses were consistent with the main results. When a Cox proportional hazards model was compared to a ROC curve analysis, all models had a slightly higher discrimination using Cox compared to ROC, but how the models performed relative to each other remained the same (Additional File 3: Figure S2). The two best performing models were still the Aleksandrova [30] and Ma Cox [12] models which both had C-statistics of 0.71 [95% CI 0.70–0.72]. The aspirin variable was only available for a subsection of the population, and when the aspirin variable was removed, the AUC slightly dropped in Hong [27] but remained the same for Imperiale [26] (Hong AUC dropped from 0.69 [95% CI 0.67–0.71] to 0.68 [0.67–0.69] and Imperiale AUC remained the same at 0.60 [0.58–0.63]).

## Discussion

In this study of CRC model performance assessed in a large prospective cohort study in China, performance of published risk models based on demographic and easily obtainable lifestyle variables varied substantially but models had good overall discrimination. Of the nine models assessed, the Ma Cox [12] and Aleksandrova [30] models had the highest discrimination (AUC 0.70), which is similar to that of the derivation cohort in the published articles (0.70 and 0.71 for the Ma and Aleksandrova models, respectively). The data from this study show that using these models, the 10% with the highest risk would include approximately one-quarter of people that go on to develop CRC. The 25% with the highest risk would include approximately half of those that develop CRC. These models were better at predicting incident CRC than simply using an age cut-off of 56 years, which had an AUC of 0.65. This study showed that several existing models could offer better efficacy in identifying those at higher risk of CRC than using age 56 as the only criteria.

The finding that most models performed better in males than in females in this Chinese population is consistent with external validations of CRC models in other populations [32]. This could relate to a difference in reporting of risk factors or a difference in the aetiology of CRC between males and females. For example, more females present with right sided colon cancer than males and differences in the association between dietary factors (such as meat and fibre consumption) and CRC by sex has been reported [20, 33]. Moreover, female hormonal factors are likely to contribute to differences in risk. Previous and current hormone replacement therapy (HRT), especially combined oestrogen-progesterone therapy, is associated with a lower risk of CRC [34, 35]. However, because fewer females in China are on HRT compared to in “western” populations, it is not clear if exogenous hormonal factors are significant contributors to developing CRC [36].

The finding that models developed in Chinese populations did not perform better than those developed in European or North American populations was unexpected but could be related to the small number of CRC cases that the models were based on, limiting their generalisability. The two Chinese models were developed based on 138 and 353 cases of CRC, compared to > 1000 cases for the Hong [27] model and > 3000 for the Aleksandrova [30] model, two of the best performing models. While other studies have shown that country-specific risk models can perform better due to differences in the distribution and impact of risk factors, this study’s findings suggest that models based on a large number of CRC cases may have more generalisability across different populations than country-specific models based on small populations.

To address whether lifestyle information is important for absolute risk beyond age alone, we assessed model performance in younger (< 56 years) and older (56 years and older) age groups. The results showed that, for most models, discrimination was higher at younger ages. Although we cannot be certain why models performed better in younger populations, it is possible that earlier onset CRC is more likely to have a heritable component where the risk factors play a bigger part in the disease pathogenesis. This finding highlights the potential utility of using risk prediction models for screening high-risk younger participants, who could be motivated to change behaviours that may influence risk over decades. Moreover, three models that included age and other medical history and lifestyle variables had a higher discrimination than the UK age cut-off model for predicting CRC risk, highlighting the relevance of modifiable ‘lifestyle’ factors to CRC risk prediction beyond age alone.

Discrimination analyses were performed using the AUC, a standard measure of discrimination, which allowed for comparison to model validation in other populations (using a C-statistic produced similar results). Compared to the discrimination of risk models for other cancers, the discrimination of the best performing models was only slightly lower than risk models for breast (0.72–0.76), melanoma (0.70–0.79), and kidney cancer (> 0.70) [37–39]. The AUC results from this study were similar but slightly lower than those from the internal validation studies for the Driver, Imperiale, Hong, Guo, and Chen models. The published Ma Cox article was the only one that performed a validation in an external population in Japan and the AUC was lower than in this study (CKB AUC was 0.70 [0.69–0.72] compared to external validation AUC of 0.64 [0.61–0.67]). A similar study externally validated risk prediction models for CRC in UK Biobank (UKB), the largest population-based cohort in the UK [32, 40]. Three models that were used in this study (Driver, Ma Point, Ma Cox) were also externally validated in UKB and they performed similarly in both cohorts (Additional File 2: Table S2).

This was the first study to externally validate multiple risk prediction models in the China Kadoorie Biobank (CKB), a large prospective cohort of 0.5 million participants from geographically diverse areas. The strengths of CKB include a prospective design, a large and diverse study population, large numbers of CRC cases by sex and by anatomical site, wider regional variation than studies used for existing scores, completeness of data, linkage to national cancer registries, and over 10 years of follow-up. A strength of this study is the direct comparison of multiple models in the same population and the re-calibration of five models to better predict risk in a Chinese population. A limitation of this study is that models were excluded from the systematic review that required family history of CRC or genetic information, as these details were not available for the main study population. Although family history of CRC may be a useful risk factor in the absence of other information, it may not be a main contributor to risk if other lifestyle information is available [26, 41]. In addition, including genetic information to risk scores for CRC has been shown to improve discrimination and calibration, but reduces their generalisability [42–44]. In a country like China, without a national screening programme, having a risk model based on easily obtainable data (like age, smoking status, BMI, alcohol consumption) could be preferable.

Future work should explore how risk models can help make recommendations about type of screening that should be offered to those at high risk, the age to begin screening, and screening intervals. An ongoing randomised controlled trial in China (TARGET-C) is comparing the effectiveness and cost-effectiveness of three different screening strategies in adults aged 50–74 (one-time colonoscopy, annual FIT, and annual risk score screening) [45]. Interim analysis has shown a high participation rate for risk-score screening and its diagnostic yield was superior to that of FIT [46]. The outcome of this trial, and others like it, will provide valuable information about the feasibility of obtaining risk factor data for use in risk scores and the potential benefits of incorporating a risk-based stratification approach along with other methods of screening into clinical practice.

## Conclusions

In conclusion, this was the first study to externally validate risk prediction models for CRC in the China Kadoorie Biobank, a large, geographically diverse prospective cohort study in China. Nine risk models were found to have good discrimination; the two best performing models included easily obtainable variables based on demographic, medical history, and lifestyle information. This study showed that three models had a higher discrimination than using the UK CRC screening age cut-off of 56. Five models have been recalibrated to better fit a Chinese population. These should be evaluated alongside other screening modalities (such as colonoscopy and FIT) to establish how these risk scores can be used to identify high risk individuals and improve screening uptake in China.

## Supplementary Information

**Additional file 1: Page S1.** Systematic review search strategy for colorectal cancer risk models. **Page S2.** TRIPOD checklist for colorectal cancer risk models. **Page S3.** Ascertainment of anthropometric measurements, covariates, and alcohol intake in the China Kadoorie Biobank. **Page S4.** Derivation of colorectal cancer risk model variables in the China Kadoorie Biobank, and **Page S5.** Full equations of the colorectal cancer risk models used for external validation in the China Kadoorie Biobank.

**Additional file 2: Table S1.** Recalibration of five colorectal cancer risk prediction models to the China Kadoorie Biobank, and **Table S2.** Comparison of three colorectal risk models validated in UK Biobank and China Kadoorie Biobank.

**Additional file 3: Figure S1.** Recalibrated curves of observed and expected 10-year risk of colorectal cancer in men and women, and **Figure S2.** Discrimination of colorectal cancer models comparing ROC curve analysis and cox regression.

## Data Availability

The China Kadoorie Biobank (CKB) is a global resource for the investigation of lifestyle, environmental, blood biochemical, and genetic factors as determinants of common diseases [14]. The CKB study group is committed to making the cohort data available to the scientific community in China, the UK, and worldwide to advance knowledge about the causes, prevention, and treatment of disease. For detailed information on what data is currently available to open access users and how to apply for it, visit: http://www.ckbiobank.org/site/Data+Access. Researchers who are interested in obtaining the raw data from the China Kadoorie Biobank study that underlines this paper should contact ckbaccess@ndph.ox.ac.uk. A research proposal will be requested to ensure that any analysis is performed by bona fide researchers and—where data is not currently available to open access researchers—is restricted to the topic covered in this paper.

## Abbreviations

AUC: Area under the receiver operator curve
BMI: Body mass index
China CDC: China National Centre for Disease Control and Prevention
CKB: China Kadoorie Biobank
CRC: Colorectal cancer
DSP: Disease Surveillance Points system
FIT: Faecal immunochemical test
HRT: Hormone replacement therapy
ICD: International Classification of Disease
ROC: Receiver operating characteristic curve
UK: United Kingdom
UKB: UK Biobank
