# Supplementary material for: External validation of models for predicting risk of colorectal cancer using the China Kadoorie Biobank
Source: BMC Med. 2022 Sep 8;20:302. doi: 10.1186/s12916-022-02488-w (PMC9454206; doi:10.1186/s12916-022-02488-w)
Supplement: Supplementary file 1 — Additional file 1: Page S1. Systematic review search strategy for colorectal cancer risk models. Page S2. TRIPOD checklist for colorectal cancer risk models. Page S3. Ascertainment of anthropometric measurements, covariates, and alcohol intake in the China Kadoorie Biobank. Page S4. Derivation of colorectal cancer risk model variables in the China Kadoorie Biobank, and Page S5. Full equations of the colorectal cancer risk models used for external validation in the China Kadoorie Biobank. [file 12916_2022_2488_MOESM1_ESM.zip › Additional File 1_Page S1.docx]

Systematic review search strategy for colorectal cancer risk models

**Medline** (Ovid MEDLINE® Epub Ahead of Print, In-Process & Other Non-Indexed Citations, Ovid MEDLINE® Daily and Ovid MEDLINE®) 1946 to present

1 exp Risk Factors/ or risk*.mp. or exp Risk/ or exp Risk Assessment/ 2962011

2 chance*.mp. 89975

3 likelihood*.mp. or exp Probability/ 1590380

4 1 or 2 or 3 3267794

5 predict*.mp. 1828132

6 exp "Early Detection of Cancer"/ 29442

7 exp models, statistical/ 429798

8 exp Models, Statistical/ or model*.mp. 4162085

9 Score*.mp. 1055900

10 5 or 6 or 7 or 8 or 9 6073051

11 exp Colorectal Neoplasms/ 212779

12 ((colorectal or rect* or colon*) adj3 (cancer* or neoplas* or tumour* or tumor*)).ti,ab. 202779

13 11 or 12 278627

14 4 and 10 and 13 25419

15 Review/ 2838461

16 Comment/ 921014

17 Letter/ 1145910

18 Editorial/ 575825

19 (((Risk Factors or risk* or Risk or Risk Assessment or chance* or (likelihood* or Probability)) and (predict* or "Early Detection of Cancer" or models, statistical or (Models, Statistical or model*) or Score*) and (Colorectal Neoplasms or ((colorectal or rect* or colon*) adj3 (cancer* or neoplas* or tumour* or tumor*)))) not (Review or Comment or Letter or Editorial)).af. 17456

20 limit 19 to (english language and humans and yr="2016 - Current") 5563

**Embase**

Embase 1974 to present

1 exp cancer risk/ or risk*.mp. or exp risk/ or exp risk factor/ or exp risk assessment/ 4326169

2 chance*.mp. 129993

3 exp probability/ or likelihood*.mp. 323151

4 1 or 2 or 3 4636518

5 exp mathematical model/ or model*.mp. or exp model/ 5414943

6 exp prediction/ or predict*.mp. 2440717

7 score.mp. 1148879

8 5 or 6 or 7 7787393

9 ((colorectal or rect* or colon*) adj3 (cancer* or neoplas* or tumour* or tumor*)).ti,ab. 298640

10 exp colon tumor/ 346771

11 exp rectum tumor/ 278568

12 exp colorectal cancer/ 196821

13 9 or 10 or 11 or 12 436387

14 4 and 8 and 13 33749

15 review.pt. 2765653

16 letter.pt. 1183437

17 editorial.pt. 697764

18 15 or 16 or 17 4646854

19 (((cancer risk or risk* or risk or risk factor or risk assessment or chance* or (probability or likelihood*)) and (mathematical model or model* or model or (prediction or predict*) or score) and (((colorectal or rect* or colon*) adj3 (cancer* or neoplas* or tumour* or tumor*)) or colon tumor or rectum tumor or colorectal cancer)) not (review or letter or editorial)).af. 31517

20 limit 19 to (human and english language and yr="2016 -Current") 13998

Prisma figure

Medline 5563

Embase 13998
