# Supplementary material for: External validation of models for predicting risk of colorectal cancer using the China Kadoorie Biobank
Source: BMC Med. 2022 Sep 8;20:302. doi: 10.1186/s12916-022-02488-w (PMC9454206; doi:10.1186/s12916-022-02488-w)
Supplement: Supplementary file 1 — Additional file 1: Page S1. Systematic review search strategy for colorectal cancer risk models. Page S2. TRIPOD checklist for colorectal cancer risk models. Page S3. Ascertainment of anthropometric measurements, covariates, and alcohol intake in the China Kadoorie Biobank. Page S4. Derivation of colorectal cancer risk model variables in the China Kadoorie Biobank, and Page S5. Full equations of the colorectal cancer risk models used for external validation in the China Kadoorie Biobank. [file 12916_2022_2488_MOESM1_ESM.zip › Additional File 1_Page S3.docx]

Assessment of anthropometric measurements

At recruitment, standing height, weight, waist circumference (WC), hip circumference and BFP were measured in local study clinics by trained health workers with the participants wearing light clothes and no shoes. Standing height was measured to the nearest 1 mm using a manufactured instrument. WC was measured to the nearest 1 mm at the narrowest part between the lowest ribs and the highest point of the iliac crest and hip circumference at the maximum width of the buttocks, using a soft nonstretchable tape. Weight and BFP were measured using a BMI composition analyzer (TANITA TBF-300GS). BMI was calculated as weight in kilograms divided by standing height in meters squared (kg/m^2^), and waist-to-hip ratio (WHR) was calculated as WC divided by hip circumference.

### Assessment of covariates

The participants were interviewed using laptop-based questionnaires at baseline to collect data on demographic and socioeconomic statuses, smoking and alcohol consumption, physical activity, self-reported medical history and reproductive history (in women). For the present study, participants who reported to have smoked at least one cigarette daily or their equivalent for at least 6 months were defined as current regular smokers, and those who drank alcohol at least once a week were defined as current regular drinkers. Physical activity was quantified with metabolic equivalent tasks (METs) based on the type, duration and intensity of self-reported physical activity and on the time spent on sedentary activity.

Assessment of alcohol intake

Self-reported alcohol drinking patterns and indicators of problem drinking at baseline were recorded by questionnaire, with details described previously. In brief, participants were classified into: abstainers; ex-regular drinkers; reduced-intake drinkers; occasional drinkers; and current regular drinkers (i.e. drinking at least weekly in the past year). Current regular drinkers were asked further questions relating to their drinking patterns, e.g. frequency, amount consumed and experience of problem drinking indicators

| **Drinking status** | |
| --- | --- |
| Abstainers^ | Past 12 months: Never drank alcohol.  In the past: Had not drunk ≥weekly. |
| Ex-regular drinkers* | Past 12 months: Never drank alcohol.  In the past: Had drunk ≥weekly. |
| Reduced-intake drinkers* | Past 12 months: Had drunk alcohol occasionally, at certain seasons, or monthly but less than weekly.  In the past: Had drunk ≥weekly. |
| Occasional drinkers | Past 12 months: Had drunk alcohol occasionally, at certain seasons, or monthly but less than weekly.  In the past: Had not drunk ≥weekly. |
| Current regular drinkers | Past 12 months: ≥Weekly (i.e., drank alcohol most weeks).  In the past: --N/A |
