# Supplementary material for: External validation of models for predicting risk of colorectal cancer using the China Kadoorie Biobank
Source: BMC Med. 2022 Sep 8;20:302. doi: 10.1186/s12916-022-02488-w (PMC9454206; doi:10.1186/s12916-022-02488-w)
Supplement: Supplementary file 1 — Additional file 1: Page S1. Systematic review search strategy for colorectal cancer risk models. Page S2. TRIPOD checklist for colorectal cancer risk models. Page S3. Ascertainment of anthropometric measurements, covariates, and alcohol intake in the China Kadoorie Biobank. Page S4. Derivation of colorectal cancer risk model variables in the China Kadoorie Biobank, and Page S5. Full equations of the colorectal cancer risk models used for external validation in the China Kadoorie Biobank. [file 12916_2022_2488_MOESM1_ESM.zip › Additional File 1_Page S4.docx]

Derivation of colorectal cancer risk model variables in the China Kadoorie Biobank

Variables used consistently across all models

**Age**: Participant age when they attended the CKB baseline survey was used in all models as a categorical or continuous variable.

**BMI**: BMI measurement was calculated from height and weight measured during the initial visit to the CKB assessment centre and was used in all models as a categorical or continuous variable.

**Sex:** Self-reported sex recorded on the baseline questionnaire was used to categorise participants into male and female in the models.

**Diabetes**: Participants were categorized as having diabetes if they had reported that they had ever received a diabetes diagnosis when attending the CKB baseline survey and not having diabetes otherwise.

Driver 2007 (USA)

**Smoking status**: Participants that reported they were occasional smokers or smokers were included as smokers, whereas ex-regular smokers and never smokers were grouped together as non-smokers for the Driver model.

**Alcohol intake**: Never, ex-regular, occasional, monthly, reduced intake drinkers were all considered to drink less than once a week, whereas weekly drinkers were considered to drink >once/week.

Ma 2010 (Japan) – point score

**Smoking status**: Never smokers, occasional smokers, and ex regular smokers were considered non-smokers or former smokers for the Ma point model, and current smokers otherwise.

**Alcohol intake**: Never regular, ex-regular, occasional, monthly, and reduced intake drinkers were all considered never or occasional drinkers for the Ma point model, whereas weekly drinkers were divided into those who drank less than 300g per week and those than drank 300g of more of alcohol.

**Physical activity**: Physical activity was defined in terms of MET-hours and split into a categorical variable based on whether the participants did less than 24.7 MET-h/day or more than or equal to 24.7 MET-h/day.

Ma 2010 (Japan) – Cox model

**Physical activity**: Physical activity was defined in terms of MET-hours and used as a continuous variable.

**Alcohol intake**: Weekly alcohol drinkers were divided into a categorical variable based on whether they consumed less than 300 grams per week or whether they consumed more than or equal to 300 grams per week.

Guo 2019 (China)

**Alcohol intake**: Never drinkers were considered non-drinkers, whereas ex-regular, occasional, monthly, reduced intake drinkers were all considered to be drinkers.

**Waist circumference:** Waist circumference was split into a categorical variable divided into those whose waist was less than 95cm and those whose waist was equal to or above 95cm.

**Occupational sitting time**: There did not exist a question on occupational sitting time in the CKB questionnaire, therefore hours of MET work per day was used to approximate occupational sitting time by assuming that participants were sitting when they were not engaged in occupational activity.

Chen 2014 (China)

**Coronary heart disease (CHD)**: Participants were considered to have CHD if they had reported a prior CHD diagnosis at baseline and considered not to have CHD otherwise.

**Egg intake:** Eating eggs daily, 4-6 days per week, or 1-3 days per week were considered frequent consumption, whereas monthly and never/rarely were considered occasional consumption.

**Defecation frequency**: Participants defecating more than once on most days and about daily were considered to be defecating once or more every day, whereas defecating once every 2-3 days and less than 3 times per week were considered to be once every 2 or more days.

Aleksandrova 2021 (Germany)

**Smoking**: Participants that self-reported as occasional smokers, on most days, and daily or almost every day smokers were considered to be smokers, whereas participants that reported they did not smoke at baseline were considered to be non-smokers.

**Physical activity**: Participants that self-reported to be exercising 1-3 times/month, 1-2 times/week, 3-5 times/week, or daily or almost every day were considered to be physically active, whereas those that reported they never or almost never exercised were not considered to be physically active.

**Fruit consumption**: A daily serving of vegetables was considered to be 100g (1). Daily vegetable intake was 700g/week or 100g/day. Consuming vegetables 4-6 days per week was considered to be 500g/week, consuming 1-3 days per week was considered 200g/week, consuming monthly was considered to be 100g/week, and never or rarely was considered to be 0g/week.

**Dairy consumption**: A daily serving of dairy was considered to be 100g.^1^ Daily dairy intake was 700g/week or 100g/day. Consuming dairy 4-6 days per week was considered to be 500g/week, consuming 1-3 days per week was considered 200g/week, consuming monthly was considered to be 100g/week, and never or rarely was considered to be 0g/week.

**Meat consumption**: A daily serving of meat was considered to be 50g. Daily meat intake was 350g/week or 50g/day. Consuming dairy 4-6 days per week was considered to be 250g/week, consuming 1-3 days per week was considered 100g/week, consuming monthly was considered to be 50g/week, and never or rarely was considered to be 0g/week.

Imperiale 2021 (USA)

**Married**: Participants that were alone in a household were considered to be living alone, participants that were living in a household with two or more people were considered to be married or living with their partner.

**Education**: Participants that went to university were considered college graduates, participants that had no formal school, or up to primary school, middle school, high school, or technical school/college were considered to be everything else.

**Significant alcohol**: CKB data is recorded in amount of alcohol consumed per week in grams. This was first converted into average number of drinks per week, by assuming one drink contained 10 grams.^2 3^

**Metabolic syndrome:** A diagnosis of metabolic syndrome requires three or more of the following: a waistline of 40 inches for men or 35 inches for women; a blood pressure of 130/85 mmHg or higher or are taking blood pressure medications; a triglyceride level above 150 mg/dl; a fasting blood glucose (sugar) level greater than 100 mg/dl or are taking glucose-lowering medications; a high-density lipoprotein level (HDL) less than 40 mg/dl (men) or under 50 mg/dl (women). All of these variables could be easily adapted in CKB, other than the glucose measurement. Fasting glucose levels were not measured among all participants in CKB, therefore we only included participants were on glucose-lowering medications in this definition. HDL levels were also not available on all participants, so that condition was not included in the definition.

**Red meat consumption:** If meat was consumed daily they were assigned 7 points; if consumed 4-6 days per week they were assigned 5 points; if consumed 1-3 days per week they were assigned 2 points; if they consumed meat monthly they were assigned 1 point; if they never/rarely ate meat they were assigned 0 points.

**Regular activity:** If participants exercised never or almost never or 1-3 times per month, they were assigned 0 points; if they exercised for 1-2 times per week, 3-5 times per week, or daily or almost every day, they were assigned -1 points.
