# Supplementary material for: External validation of models for predicting risk of colorectal cancer using the China Kadoorie Biobank
Source: BMC Med. 2022 Sep 8;20:302. doi: 10.1186/s12916-022-02488-w (PMC9454206; doi:10.1186/s12916-022-02488-w)
Supplement: Supplementary file 1 — Additional file 1: Page S1. Systematic review search strategy for colorectal cancer risk models. Page S2. TRIPOD checklist for colorectal cancer risk models. Page S3. Ascertainment of anthropometric measurements, covariates, and alcohol intake in the China Kadoorie Biobank. Page S4. Derivation of colorectal cancer risk model variables in the China Kadoorie Biobank, and Page S5. Full equations of the colorectal cancer risk models used for external validation in the China Kadoorie Biobank. [file 12916_2022_2488_MOESM1_ESM.zip › Additional File 1_Page S5.docx]

Full equations of the colorectal cancer risk models used for external validation in the China Kadoorie Biobank

**Driver 2007 (USA):**

age: 2 points if 50-59; 4 points if 60-69; 6 points if ≥70.

BMI: 1 point if 25-29.9; 2 points if ≥30.

smoking status: 1 point if smoker, 0 points if non smoker.

alcohol intake: 1 point if ≥once/week; 0 points if less.

**Ma 2010 (Japan) point score:**

age: 0 points if 40-44; 1 point if 45-49; 3 points if 50-54; 4 points if 55-59; 5 points if 60-64; 6 if 65-69.

BMI:0 points if <25kg/m2; 1 point if ≥25kg/m2.

smoking status: 0 points if non-smoker or former smoker; 1 point if current smoker.

alcohol intake: 0 points if never or occasional; 1 point if regular <300g/week; 2 points if regular ≥300g/week.

physical activity score: 0 points if <24.7 MET-h/day; -1 if ≥24.7 MET-h/day.

**Ma 2010 (Japan) cox proportional hazard model:**

The probability of developing colorectal cancer was calculated as 1- S(t)exp(f[x,M]); Where

S(t) = 0.9882

f(x,M) = ((0.080 * (individual’s age – 52.02)) + ((0.04 * (individual’s BMI – 23.658)) + ((-0.019 * (individual’s physical activity – 21.08)) + (0.071 * (1 (if ex- smoker, else 0) – 0.05953)) + (0.239 * (1 (if current smoker, else 0) – 0.264)) + (-0.163 * (1 (if never drinks alcohol, else 0) – 0.458)) + (0.358 * 1 (if regular alcohol consumption <300g/w, else 0) – 0.1016)) + (0.659 * (1 (if regular alcohol consumption ≥300g/w, else 0) – 0.0468))

**Guo 2019 (China):**

age: 0 points if < 40 years; 6 points if 40-49; 8 points if 50-59; 11 points if ≥60.

alcohol consumption: 0 points if non drinker; 2 points if drinker.

waist circumference: 0 points if <95cm; 1 point if ≥95cm.

occupational sitting time (hr/day): 0 points if >8 h/d; 3 points if 4-8 h/d; 4 ponts if <4 h/d.

diabetes: 0 points if non diabetic; 1 point if diabetic.

**Chen 2014 (China):**

age score: 0 points if 40-49; 1 point if 50-59; 2 points if 60-69; 3 points if >69.

sex: 0 points if female; 2 points if male.

Coronary heart disease: 0 points if no coronary heart disease; 3 points if yes

egg intake: 0 points if frequently; 1 point if occasionally.

defecation frequency: 0 points if once or more every day; 1 point if once every 2 or more days.

**Betes 2003 (Spain):**

age: 0 points if <= 50 years; 1 point if 51-60 years; 2 points if 61-70; 3 points if 71-80; 4 points if >80.

sex: 0 if female; 2 if male.

BMI: 0 points if <=25; 1 point if 25-35; 2 points if >35.

**Aleksandrova 2021 (Europe):**

Absolute risk -colorectal cancer within 10 years.

1 − 0.9943exp(Risk Scorei − 6.8089)

Risk Scorei =

0.0781 x Age(years)

+ 0.0117 x Waist circumference(cm)

+ 0.0115 x Body height(cm)

+ 0.1292 x Daily alcohol (yes = 1, no = 0)

+ 0.2125 x Smoking (yes = 1, no = 0)

- 0.0964 x Physically active (yes = 1, no = 0)

− 0.0773 x Vegetable intake (per 100g per day)

- 0.0166 x Dairy product intake (per 100g per day)

+ 0.0808 x Processed meat intake (per 50g/day)

+ 0.0268 x Sugar and confectionary (per 50g/day)

**Imperiale 2021 (USA):**

age: 0 points for age 50-54; 1 point for 55-59; 2 points for 60-64; 3 points for 65-69; 4 points for age >= 70

gender: 0 points for women; 3 points for men

married: 0 points for living alone; -3 for married/living with partner

education: 0 points for everything else; -1 points for college graduate

smoking: 0 points for 0-<30 pack years; 3 points for >= 30 pack years

significant etoh: 0 if less than 11 drinks for men and 6 drinks for women; 1 point for men >11/week women >6/week

NSAID: 0 points for less than daily and >= 1 year; -4 points for daily use and >= 1 year

metabolic syndrome: 1 point if >= 3 AJCC criteria

red meat consumption per week: 0-9 points based on number of servings per week; 9 points for >= 9 servings

regular activity: 0 points for less than than once a week for 2 or more years; -1 point for more than once a week for 2 or more years

moderate activity (over the last year): 0 points for 0-1.6 hours/week; -1 point for 1.7-4.9 hours/week; -2 points for >= 5 hours/week

vigorous activity: 0 points for < 1 hour/week for last 12 months; -2 points for >= 1 hour/week for last 12 months

aspirin use: 0 points for less than daily use for < 1 year; -2 for daily use and >= 1 year
