# Supplementary material for: External validation of models for predicting risk of colorectal cancer using the China Kadoorie Biobank
Source: BMC Med. 2022 Sep 8;20:302. doi: 10.1186/s12916-022-02488-w (PMC9454206; doi:10.1186/s12916-022-02488-w)
Supplement: Supplementary file 2 — Additional file 2: Table S1. Recalibration of five colorectal cancer risk prediction models to the China Kadoorie Biobank, and Table S2. Comparison of three colorectal risk models validated in UK Biobank and China Kadoorie Biobank. [file 12916_2022_2488_MOESM2_ESM.zip › Additional File 2_Table S1.docx]

Recalibration of five colorectal cancer risk prediction models to the China Kadoorie Biobank

Ma point risk score

| Total score | 10-year risk (%) | |
| --- | --- | --- |
|  | Uncalibrated | Re-calibrated to CKB |
| -1 | 0.2 | 0.199 |
| 0 | 0.3 | 0.235 |
| 1 | 0.5 | 0.308 |
| 2 | 0.7 | 0.380 |
| 3 | 0.9 | 0.452 |
| 4 | 1.3 | 0.597 |
| 5 | 1.8 | 0.778 |
| 6 | 2.4 | 0.994 |
| 7 | 3.3 | 1.320 |
| 8 | 4.6 | 1.789 |
| 9 | 5.9 | 2.259 |
| 10 | 7.4 | 2.801 |

Driver score

| Total score | 20-year risk (%) | 10-year risk (%) | |
| --- | --- | --- | --- |
|  | Uncalibrated | Uncalibrated | Re-calibrated to CKB |
| 0 | 0.006 | 0.3 | 0.235 |
| 1 | 0.009 | 0.5 | 0.308 |
| 2 | 0.009 | 0.7 | 0.380 |
| 3 | 0.014 | 0.9 | 0.452 |
| 4 | 0.022 | 1.3 | 0.597 |
| 5 | 0.032 | 1.8 | 0.778 |
| 6 | 0.044 | 2.4 | 0.994 |
| 7 | 0.046 | 3.3 | 1.320 |
| 8 | 0.065 | 4.6 | 1.789 |
| 9 | 0.086 | 5.9 | 2.259 |
| 10 | 0.086 | 7.4 | 2.801 |

Guo score

| Total score | 10-year risk (%) | |
| --- | --- | --- |
|  | Uncalibrated | Re-calibrated to CKB |
| 0-5 | 0.03 | 0.2019 |
| 6-10 | 0.12 | 0.3240 |
| 11-15 | 0.45 | 0.7719 |
| 16-19 | 1.04 | 1.5725 |

Imperiale score

| Total score | 10-year risk (%) | |
| --- | --- | --- |
|  | Uncalibrated | Re-calibrated to CKB |
| -13 to -5 (Low risk) | 1.50 | 0.5834 |
| -4 to -2 (Intermediate risk) | 7.06 | 0.8056 |
| >= 3 (High risk) | 27.3 | 1.6144 |

Hong score

| Decile of predicted risk | 10-year risk (%) | |
| --- | --- | --- |
|  | Uncalibrated | Re-calibrated to CKB |
| 1 | 0.260 | 0.1816 |
| 2 | 0.520 | 0.2385 |
| 3 | 0.937 | 0.3297 |
| 4 | 1.145 | 0.3752 |
| 5 | 1.197 | 0.3866 |
| 6 | 1.509 | 0.4549 |
| 7 | 2.445 | 0.6597 |
| 8 | 2.653 | 0.7052 |
| 9 | 4.214 | 1.0468 |
| 10 | 5.962 | 1.4293 |
