# Supplementary material for: External validation of models for predicting risk of colorectal cancer using the China Kadoorie Biobank
Source: BMC Med. 2022 Sep 8;20:302. doi: 10.1186/s12916-022-02488-w (PMC9454206; doi:10.1186/s12916-022-02488-w)
Supplement: Supplementary file 2 — Additional file 2: Table S1. Recalibration of five colorectal cancer risk prediction models to the China Kadoorie Biobank, and Table S2. Comparison of three colorectal risk models validated in UK Biobank and China Kadoorie Biobank. [file 12916_2022_2488_MOESM2_ESM.zip › Additional File 2_Table S2.docx]

**Supplementary Table S2: Comparison of CRC model performance in the China Kadoorie Biobank (CKB) and the UK biobank (UKB) for three models (Driver, Ma Point, Ma Cox).** The Driver model was only evaluated in males in UKB; the Ma models were evaluated in both males and females in both cohorts.

|  | **UK Biobank**  (1718 CRC cases) | **China Kadoorie Biobank**  (2976 CRC cases) |
| --- | --- | --- |
| **Driver model** | | |
| Males | 0.67 [95% CI 0.66-0.69] | 0.61 [0.59-0.63] |
| **Ma Point model** | | |
| Males | 0.68 [0.67-0.70] | 0.66 [0.64-0.67] |
| Females | 0.64 [0.63-0.66] | 0.64 [0.63-0.66] |
| **Ma Cox model** | | |
| Males | 0.69 [0.68-0.71] | 0.70 [0.69-0.72] |
| Females | 0.64 [0.62-0.66] | 0.69 [0.68-0.70] |
