# Supplementary figures and images for: External validation of models for predicting risk of colorectal cancer using the China Kadoorie Biobank
Source: BMC Med. 2022 Sep 8;20:302. doi: 10.1186/s12916-022-02488-w (PMC9454206; doi:10.1186/s12916-022-02488-w)

(A)

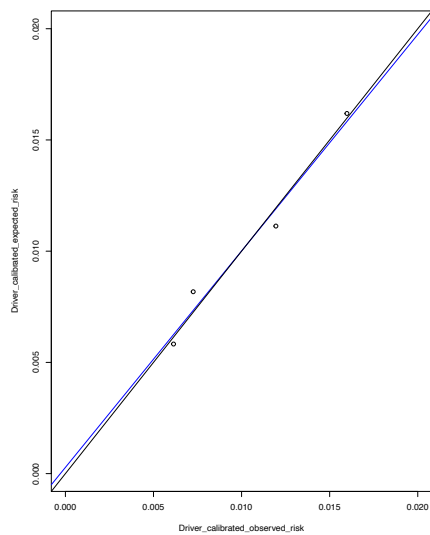

(B)

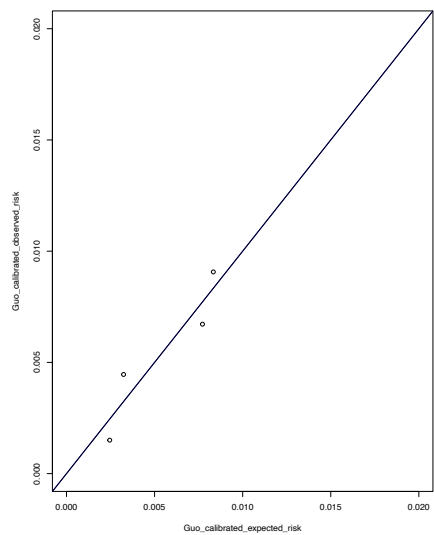

(C)

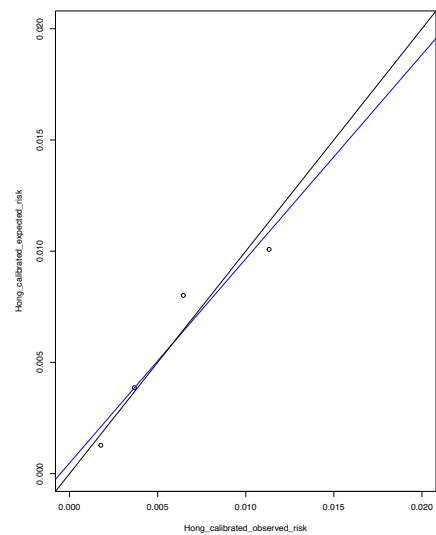

(D)

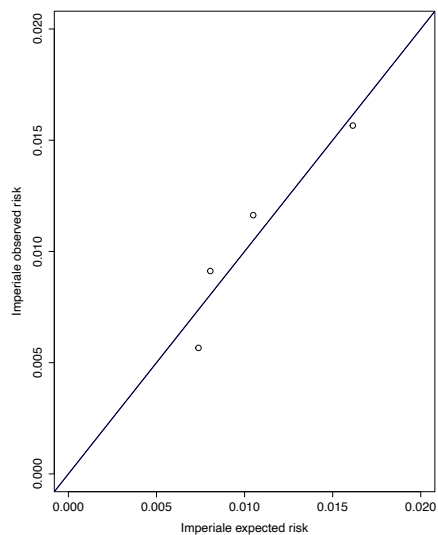

(E)

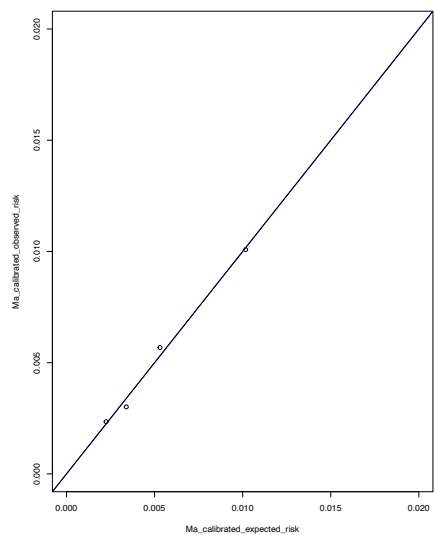

Supplement: Supplementary file 3 — Additional file 3: Figure S1. Recalibrated curves of observed and expected 10-year risk of colorectal cancer in men and women, and Figure S2. Discrimination of colorectal cancer models comparing ROC curve analysis and cox regression. [file 12916_2022_2488_MOESM3_ESM.zip › Additional File 3_Figure S1.pdf]

**C statistic (95% CI)**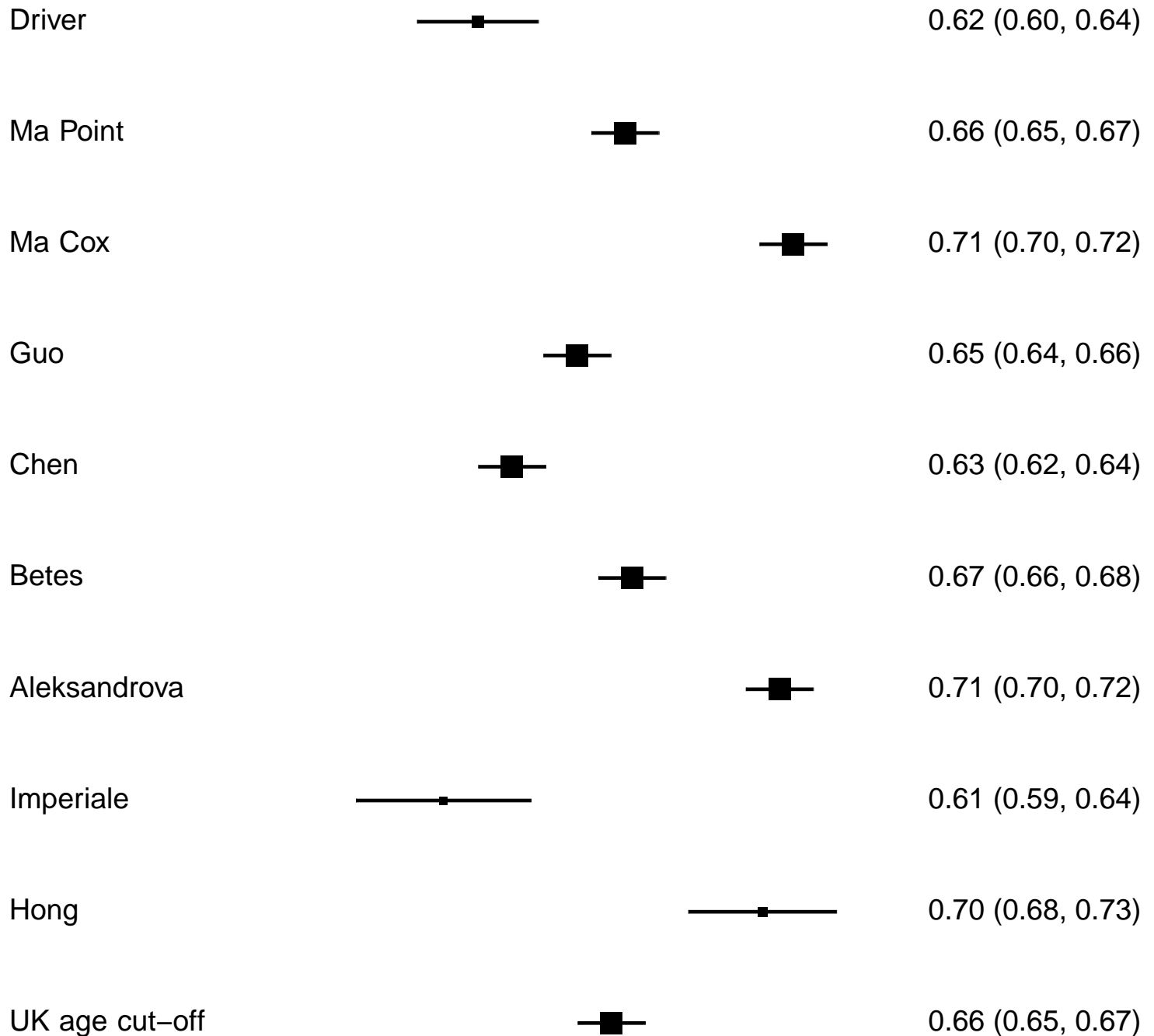

0.55 0.60 0.65 0.70 0.75

**C statistic**

Supplement: Supplementary file 3 — Additional file 3: Figure S1. Recalibrated curves of observed and expected 10-year risk of colorectal cancer in men and women, and Figure S2. Discrimination of colorectal cancer models comparing ROC curve analysis and cox regression. [file 12916_2022_2488_MOESM3_ESM.zip › Additional File 3_Figure S2.pdf]
